# The association between weight-adjusted-waist index and psoriasis: A cross-sectional study based on NHANES 2009 to 2014

**DOI:** 10.1097/MD.0000000000040808

**Published:** 2024-12-06

**Authors:** Yanan Tuo, Junchen He, Tao Guo

**Affiliations:** a Department of Dermatology, Tianjin Academy of Traditional Chinese Medicine Affiliated Hospital, Tianjin, China.

**Keywords:** metabolism, NHANES, obese, psoriasis, weight-adjusted-waist index

## Abstract

Weight-adjusted-waist index (WWI) is an anthropometric indicator of central obesity, which is calculated by dividing the waist circumference (WC) by the squared weight. The purpose of this study was to investigate the association between WWI and psoriasis in adults. Multivariate logistic regression and smoothing curve fitting were used to investigate the relationship between WWI and psoriasis based on data from the National Health and Nutrition Examination Survey (NHANES) 2009 to 2014. Subgroup analysis and interaction tests were employed to examine the population-level stability of this connection. There was a positive association between WWI and psoriasis in 15,932 participants > 20 years of age. In the fully adjusted model, each 1-unit increase in WWI was associated with a 14% increase in the risk of developing psoriasis [1.14 (1.01, 1.32)]. Participants in the highest quartile of WWI had a 38% higher risk of developing psoriasis than those in the lowest quartile [1.38 (1.01, 1.94)]. This positive association was more pronounced in males. WWI is positively associated with psoriasis in US adults. Our findings imply that WWI has the potential to improve psoriasis prevention in the general population.

## 1. Introduction

Psoriasis is a common inflammatory skin disease that can influence the skin and/or joints, serious disease is linked to significant impairment in physical and mental health.^[1]^ It has recently been determined that psoriasis is an inflammatory disease affecting multiple organ systems that can lead to metabolic syndrome, inflammatory bowel disease, diabetes, and cardiovascular disease.^[2,3]^ It has also been observed that the prevalence of disease is rising.^[4]^ As a result, it is imperative to identify causes that may be controlled or prevented to reduce the prevalence of psoriasis.

The number of people affected by obesity is rising, and its prevalence has reached epidemic proportions worldwide.^[5–7]^ Obesity has been associated with the onset of various diseases, including cardiovascular, diabetes, osteoarticular, immune system diseases and several malignancies.^[8–12]^ Waist circumference (WC) and body mass index (BMI) are 2 commonly used measurements for evaluating obesity. Nevertheless, it is impossible to distinguish between muscle and fat mass using such signs.^[13–15]^ According to recent research, body composition and fat distribution can be utilized to more precisely identify metabolic diseases.^[16]^ Weight-adjusted-waist index (WWI) is an anthropometric indicator of central obesity, which is calculated by dividing the WC by the squared weight.^[17]^ Even within distinct BMI categories, it could reflect components of both muscle and fat mass.^[18,19]^ WWI as a new type of obesity index, standardizes WC with weight, incorporating the strengths of WC while attenuating the relationship with BMI.^[17]^ The WWI accounts for central obesity concerns unrelated to weight in addition to differentiating between fat and muscle mass.^[18]^ According to previous studies, it outperforms BMI, body shape index (ABSI), and waist-to-height ratio (WHtR) as a notable predictor of cardiovascular morbidity and mortality.^[20]^ Furthermore, WWI is a greater predictor of incident hypertension compared to BMI and WC.^[21]^

Scientific evidence suggests that the relationship between obesity and psoriasis may be complex, with dietary practices, lifestyle choices, genetic predispositions, and the microbiome all being important in the development of both diseases due to their associations with long-term pro-inflammatory states.^[22]^ The association between WWI and psoriasis has not been investigated before despite the possibility that WWI is a sign of central obesity. It is important to explore the association of obesity evaluated by WWI and psoriasis to gain more awareness about the negative effects of obesity on psoriasis. Therefore, we aimed to investigate the association between WWI and psoriasis among the US population using data from National Health and Nutrition Examination Survey 2009 to 2014 (NHANES).

## 2. Methods

### 2.1. Study population

**Figure 1.**
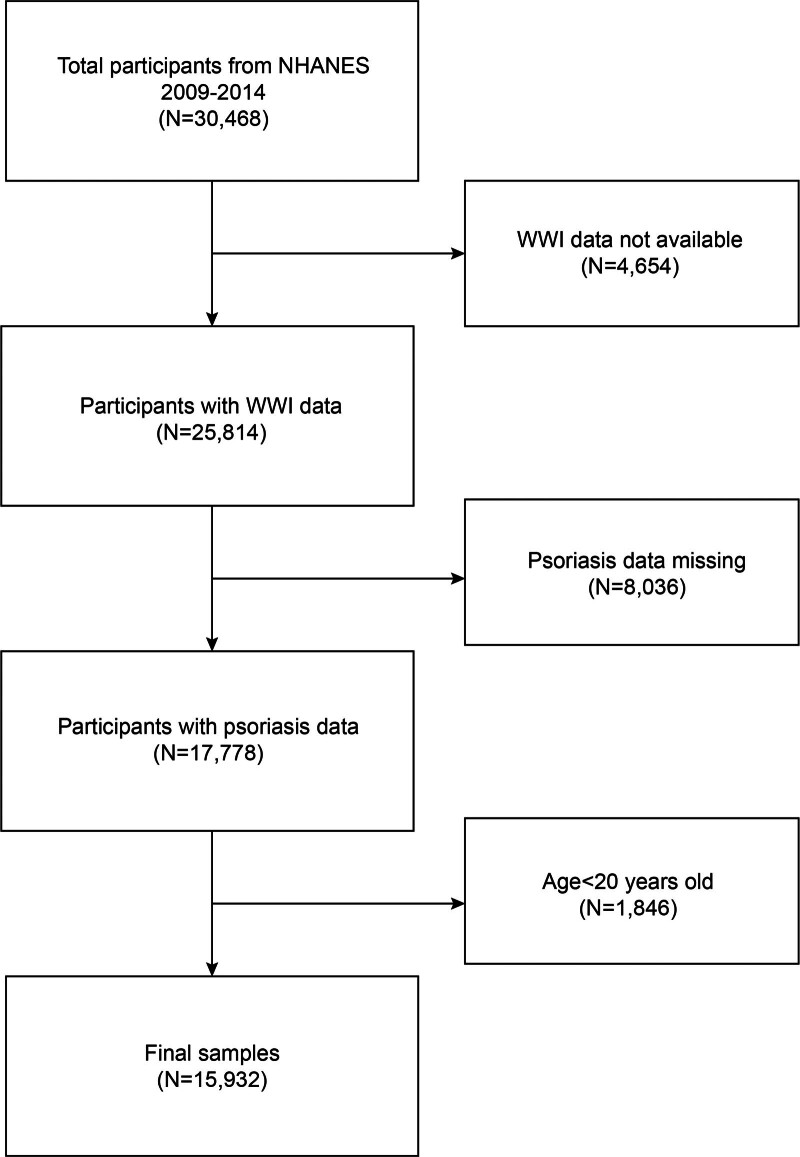
Flow chart of participants selection. NHANES = National Health and Nutrition Examination Survey.

The NHANES is a nationally representative survey conducted by the Centers for Disease Control and Prevention.^[23,24]^ The study procedure received approval from the Research Ethics Review Board of the National Center for Health Statistics (NCHS). Every participant gave written consent at the time of recruiting.^[25,26]^ The NHANES study designs and data are all available to the public at www.cdc.gov/nchs/nhanes/. Psoriasis data were only available to individuals aged 16 to 80 in the NHANES 2009 to 2014 cycles. We excluded 4654 participants without available WWI data, 8036 participants with missing psoriasis data, and 1846 participants under 20 years old. The study eventually included 15,932 participants (Fig. 1).

### 2.2. Weight-adjusted-waist index

WWI is an anthropometric index based on WC and weight to measure central obesity. In NHANES, weight and WC measurements were made by trained health technicians. Each participant’s WWI was determined by dividing their weight in kilograms squared by their WC in centimeters and rounding to 2 decimal places. (WWI = WC/Weight^2^, WC in centimeters, Weight in kilograms). Higher WWI was associated with higher levels of obesity. WWI was 1 of the exposure variables in our study.

### 2.3. Diagnosis of psoriasis

In response to the question, “Have you ever been told by a health care provider that you had psoriasis?” psoriasis was self-reported.

### 2.4. Covariables

Covariates included age, race, gender, smoking status, alcohol drinking status, coronary heart disease, high blood pressure, diabetes, low-density lipoprotein cholesterol (LDL-C), triglyceride, total cholesterol, income-to-poverty ratio (PIR), education level and BMI.

### 2.5. Statistical analysis

The participant’s demographics were evaluated by WWI quartile using the chi-square test and *t*-test. The linear associations between WWI and psoriasis were examined using weighted multivariate linear and logistic regression analyses. After WWI was transformed from a continuous variable to a categorical variable (quartile), a trend test was used to examine the linear association between WWI and psoriasis. The “*P* for trend” represents the level of significance in testing the null hypothesis. Specifically, it examines whether there is a systematic change in the outcome variable (psoriasis) as the levels of an ordered categorical variable increase (WWI). Subgroup analysis was used to investigate the association between WWI and psoriasis in people of different gender, age, and diabetes status, and interaction tests were used to investigate whether the relationships were consistent across subgroups. Smoothing curve fitting was used to explore the positive association between WWI and psoriasis.^[27]^ *R* (version 4.2) or Empowerstats (version 5.0) were used for all analyses. The definition of statistical significance is 2-sided *P* < .05.

### 2.6. Ethical statement

The portions of this study involving human participants, human materials, or human data were conducted by the Declaration of Helsinki and were approved by the NCHS Ethics Review Board. The patients/participants provided written informed consent to participate in this study.

## 3. Results

### 3.1. Baseline characteristics

Of a total of 15,932 participants older than 20 years, the mean (SD) age was 48.69 (17.49) years, with 51.20% female and 42.81% non-Hispanic White. The WWI ranges for tertiles 1 to 4 were 8.37 to 10.46, 10.47 to 11.03, 11.04 to 11.61, and 11.62 to 14.80, respectively. There was an overall prevalence of 2.75% (weighed proportion) of psoriasis and it also increased as the WWI tertile increased (tertile 1: 2.03%; tertile 2: 2.71%; tertile 3: 2.74%; tertile 4: 3.51%; *P* < .001). We observed statistically significant differences by gender, race, education, BMI, smoking, diabetes, hypertension, coronary heart disease, high blood pressure, drinking, family PIR, triglycerides, LDL-C, asthma, arthritis, thyroid problems, chronic bronchitis, and total cholesterol (all *P* < .001) among WWI tertiles (Table 1).

**Table 1:** Basic characteristics of participants by weight-adjusted-waist index among U.S. adults.

Characteristics	Weight-adjusted waist index				*P* value
	Q1 (n = 3983)	Q2 (n = 3983)	Q3 (n = 3983)	Q4 (n = 3983)	
Age (yr)	37.34 ± 14.11	46.20 ± 15.63	52.71 ± 16.17	58.51 ± 16.47	<.001
Gender, (%)					
Male	61.74	52.90	47.05	33.54	<.001
Female	38.26	47.10	52.95	66.46	
Race/ethnicity, (%)					
Mexican American	7.53	13.56	16.92	18.73	<.001
Other Hispanic	7.58	9.09	11.78	11.00	
Non-Hispanic White	42.93	41.88	41.02	45.44	
Non-Hispanic Black	27.74	20.86	19.61	16.09	
Other races	14.21	14.61	10.67	8.74	
Education level, (%)					
<high school	14.26	20.97	27.60	34.22	<.001
High school	20.54	21.24	22.72	23.88	
>high school	65.20	57.79	49.68	41.90	
Smoked at least 100 cigarettes in life, (%)					
Yes	41.01	43.56	45.49	46.82	<.001
No	58.99	56.44	54.51	53.18	
Diabetes, (%)					
Yes	2.61	6.38	14.01	24.38	<.001
No	97.39	93.62	85.99	75.62	
Coronary heart disease, (%)					
Yes	0.85	2.66	4.47	6.90	<.001
No	99.15	97.34	95.53	93.10	
High blood pressure, (%)					
Yes	15.06	29.73	41.28	54.96	<.001
No	84.94	70.27	58.72	45.04	
Family PIR	2.65 ± 1.70	2.63 ± 1.68	2.47 ± 1.64	2.12 ± 1.50	<.001
BMI	24.75 ± 4.58	27.72 ± 5.41	30.04 ± 6.18	33.21 ± 7.54	<.001
Triglyceride (mg/dL)	101.97 ± 85.91	118.36 ± 89.17	139.27 ± 156.51	148.95 ± 100.57	<.001
LDL-C (mg/dL)	107.89 ± 32.28	116.61 ± 35.06	116.64 ± 36.60	114.61 ± 35.85	<.001
Total cholesterol (mg/dL)	184.21 ± 37.90	195.62 ± 40.68	196.23 ± 42.04	195.68 ± 44.58	<.001
Had at least 12 alcohol drinks/1 yr (%)					
Yes	80.81	76.31	71.88	63.96	<.001
No	19.19	23.69	28.12	36.04	
Psoriasis, (%)					
Yes	2.03	2.71	2.74	3.51	<.001
No	97.97	97.29	97.26	96.49	
Asthma, (%)					
Yes	13.73	12.98	13.91	17.55	<.001
No	86.27	87.02	86.09	82.45	
Arthritis, (%)					
Yes	10.42	18.80	30.36	41.78	<.001
No	89.58	81.20	69.64	58.22	
Thyroid problems, (%)					
Yes	4.87	7.68	10.92	15.49	<.001
No	95.13	92.32	89.08	84.51	
Chronic bronchitis, (%)					
Yes	2.74	4.14	5.02	9.26	<.001
No	97.26	95.86	94.98	90.74	

Mean ± SD for continuous variables: the *P* value was calculated by the weighted linear regression model; (%) for categorical variables: the *P* value was calculated by the weighted chi-square test.

Abbreviations: BMI = body mass index, LDL-C = low-density lipoprotein, PIR = the ratio of income-to-poverty, Q = quartile.

### 3.2. Higher WWI in associated with psoriasis

**Figure 2.**
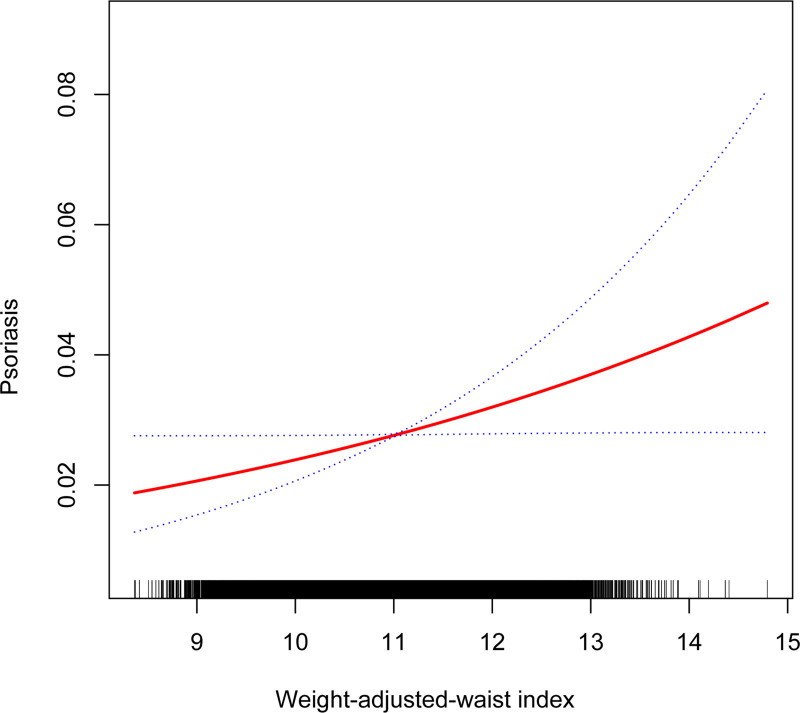
The nonlinear associations between WWI and psoriasis. The solid red line represents the smooth curve fit between variables. Blue bands represent the 95% of confidence interval from the fit. WWI = weight-adjusted-waist index.

Table 2 shows the association between WWI and psoriasis. We found higher WWI was correlated with psoriasis both in the crude model [1.28 (1.15, 1.44)] and adjusted model [1.23 (1.07, 1.40)]. After full adjustment, each unit of higher WWI score was found to be associated with a 21% increased risk of developing psoriasis (OR = 1.21, 95% CI 1.05–1.45). After WWI was classified as quartiles, the above correlation remained statistically significant (all *P* for trend < .05). In the fully adjusted model, compared with the lowest WWI tertile (quartile 1), participants in the highest WWI tertile exhibited a significantly 0.83-fold increased likelihood. (OR = 1.83, 95% CI 1.01–2.34; *P* for trend = .0214), respectively. In addition, the smoothed curve fitting results further validated the positive association between WII with psoriasis (Fig. 2).

**Table 2:** Association between weight-adjusted waist index and psoriasis.

WWI	Psoriasis	*P* for tend
	OR (95% CI)	
Crude model (model 1)		
Continuous	1.28 (1.15, 1.44)	
Categories		
Quartile 1	0 (ref)	<.0001
Quartile 2	1.34 (1.00, 1.80)	
Quartile 3	1.36 (1.01, 1.81)	
Quartile 4	1.75 (1.33, 2.32)	
Minimally adjusted model (model 2)		
Continuous	1.23 (1.07, 1.40)	
Categories		
Quartile 1	0 (ref)	.0123
Quartile 2	1.27 (0.94, 1.71)	
Quartile 3	1.25 (0.91, 1.71)	
Quartile 4	1.54 (1.12, 2.12)	
Fully adjusted model (model 3)		
Continuous	1.21 (1.05, 1.45)	
Categories		
Quartile 1	0 (ref)	.0214
Quartile 2	1.21 (0.87, 1.92)	
Quartile 3	1.76 (0.91, 1.94)	
Quartile 4	1.83 (1.01, 2.34)	

Model 1: no covariates were adjusted.

Model 2: age, gender, and race were adjusted.

Model 3: age, gender, race, education level, PIR, BMI, drinking alcohol, smoking, diabetes, coronary heart disease, high blood pressure, LDL-C, asthma, arthritis, thyroid problems, chronic bronchitis, and triglycerides were adjusted.

Abbreviations: BMI = body mass index, LDL-C = low-density lipoprotein, PIR = the ratio of income-to-poverty, Q = quartile, WWI = weight-adjusted waist index.

### 3.3. Subgroup analyses

**Figure 3.**
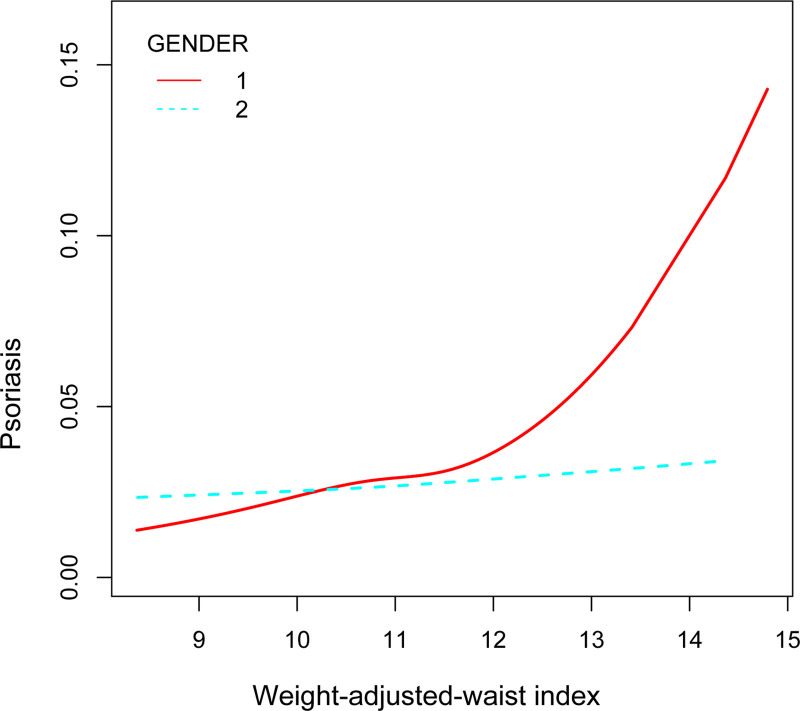
Association between WWI and psoriasis stratified by gender. WWI = weight-adjusted-waist index.

To evaluate whether the association between WWI and psoriasis was consistent in the overall population and for the potentially different population settings, we conducted subgroup analysis and interaction tests stratified by age, gender, and diabetes (Table 3). Our results showed that the associations were inconsistent. As is shown in Figure 3, we detected significant interaction for sex (*P* for interaction < .05), while there was no statistical significance for age and BMI. WWI with psoriasis remained positively associated in male, older adults greater than or equal to 60 years. Taken together, our results demonstrated the association of WWI and psoriasis showed dependence on sex, it may be appropriate for male.

**Table 3:** Subgroup analysis of the association between WWI and psoriasis.

Subgroup	Psoriasis [OR (95%CI)]	*P* for interaction
Sex		
Male	1.49 (1.26, 1.77)	.0331
Female	1.16 (0.99, 1.36)	
Age		
<60 yr	1.21 (1.04, 1.40)	.2759
≥60 yr	1.40 (1.12, 1.73)	
Diabetes, (%)		
Yes	1.66 (1.00, 2.78)	.8841
No	1.47 (1.20, 1.81)	

Age, gender, race, education level, PIR, BMI, drinking alcohol, diabetes, coronary heart disease, high blood pressure, LDL-C, asthma, arthritis, thyroid problems, chronic bronchitis, and triglycerides were adjusted.

## 4. Discussion

In the cross-sectional research that enrolled 15,932 representative participants, we observed the positive associations between WWI and psoriasis, and there was significant dependence of gender on this association, indicating that higher levels of WWI may lead to an increased risk of developing psoriasis, especially in the males. Our results suggest that WWI may have potential clinical value in the diagnosis of psoriasis risk and disease severity.

To our knowledge, this is the first study to assess the relationship between WWI and psoriasis, which emphasizes the positive association of WWI level and higher psoriasis risks. Previous studies have found that obesity plays a fundamental role in psoriasis.^[28–30]^ Owing to the widespread occurrence and significant negative effects of obesity, an increasing number of indicators are being employed to assess obesity, with a particular focus on the identified detrimental intra-abdominal fat mass. BMI, as a widely used obesity index, was found to possess a “U-shaped pattern” with all-cause mortality, with the highest risk in the most overweight and underweight participant groups.^[13,31]^ However, numerous studies proposed the “obesity paradox” phenomenon of BMI, which refers to the relatively obese participants embracing a better prognosis than those with a normal range of BMI.^[17,20,32]^ A comparable occurrence was also observed with WC when it was used in a study that focused on heart failure.^[15]^ The unanticipated paradox might be brought on by BMI and WC’s incapacity to differentiate between fat and muscle mass. WWI could be a more complete and reliable indicator of obesity.

As obesity increasingly becomes a serious global public health concern and its close link with many diseases is revealed.^[33]^ Ko et al^[30]^ evaluated ten randomized controlled trials among 1,163 participants and interventions lasting at least 12 weeks, highlighted the potential connection between psoriasis and obesity. A recent large-scale population-based Norwegian study including over 35,000 participants has found a link between metabolic syndrome and a higher chance of developing psoriasis. The examination of metabolic variables revealed that obesity plays an important part in this correlation.^[34]^ A study by Setty et al^[35]^ involved 78,626 women (of whom 892 reported having psoriasis) indicating that adiposity and weight gain were potential risk factors for the development of psoriasis. According to a recent prospective study, obesity and excessive abdominal fat mass quadrupled the incidence of psoriasis.^[28]^ According to a meta-analysis of 7 prospective studies with 17,636 participants, higher BMI, WC, WHtR, and weight gain were linked to an increased risk of psoriasis, with a 2 to 4-fold increase in the risk of psoriasis among those at the high end of each adiposity measures.^[36]^ Besides that, the association between obesity and psoriasis has been demonstrated in animal studies. It was demonstrated using an obese mouse model with imiquimod-induced psoriasiform dermatitis that obesity may acutely worsen the severity of the condition in mice.^[37]^ In our analysis, it showed a positive correlation between WWI and psoriasis in both the crude and adjusted models. This positive association was more pronounced in males. Accordingly, when faced with nutritional problems, males are more prone than females to acquire obesity, insulin resistance, and hyperglycemia when taking into account practically all animal models.^[38]^ The protective effects of endogenous estrogens are demonstrated by clinical and experimental studies, mostly due to the activation of estrogen receptor α in a variety of organs, such as the brain, liver, skeletal muscle, adipose tissue, and pancreatic beta cells. A deeper examination of the underlying mechanisms, particularly the function of sex chromosomes, fetal/neonatal programming, and epigenetic alterations, is necessary in addition to sex steroids.^[39]^

The psoriasis and obesity etiology involve alterations to the microbiota, a feature that is also shared by other chronic inflammatory diseases.^[40]^ They have a strong connection to autoimmune illnesses,^[41,42]^ which are characterized by an imbalance in lymphocyte production and an increase in IL-17 production. Furthermore, obesity modifies the inflammatory cells’ biological makeup and activity in the skin. Nakamizo et al^[43]^ reported an accumulation of γδ T cells that produce IL-17A in psoriatic skin lesions of high-fat diet (HFD)-induced obese mice, which exacerbates psoriatic dermatitis. Additionally, genetically engineered diabetic (db/db) mice demonstrated heightened psoriatic skin inflammation with enhanced levels of IL-17A and IL-22 [37]. Another study demonstrated that a prolonged HFD lasting 9 months stimulated the skin’s accumulation of specific CD11c + macrophages, in a way that was dependent on the epidermal fatty acid binding protein (E-FABP).^[44]^

The association between obesity and psoriasis has been previously well-documented in the literature, and the use of WWI in this study provides further evidence in this area. However, since NHANES is a cross-sectional study, we are unable to provide risk stratification based on WWI levels and psoriasis, as it does not follow individuals over time. One of our study’s strengths is the intricate multi-stage probability sampling design we used, which improved the study’s representativeness and dependability. Our research has several limitations. First, we were unable to determine a causal association between WWI and psoriasis because of the design of the cross-sectional analysis. Even after adjusting for some confounding variables, the results might have been affected by additional confounding variables, such as the use of steroids and diuretics. We were limited in our ability to include these covariables in our analysis since information on them was not gathered in NHANES and was not available in the original dataset. Besides that, although the diagnosis of psoriasis comes from a medical professional or health care practitioner, reliance on self-reported psoriasis conditions can lead to reporting bias and lack of clinical validation. The specific types of psoriasis were not delineated in detail, which could potentially affect the results of the study. In addition, NHANES was a US-based study, thus we could only evaluate the association between WWI and psoriasis in US adults.

## 5. Conclusion

In conclusion, our study found that WWI and psoriasis were positively correlated, and this association was more significant in males. The WWI may be of potential value in clinical practice for identifying psoriasis severity.

## Acknowledgments

We would like to thank all participants in this study.

## Author contributions

**Conceptualization:** Yanan Tuo.

**Data curation:** Yanan Tuo.

**Investigation:** Yanan Tuo.

**Methodology:** Yanan Tuo.

**Supervision:** Junchen He.

**Validation:** Junchen He.

**Writing – original draft:** Tao Guo.

**Writing – review & editing:** Tao Guo.
